# Association of the TNF-α-308, TNF-α-238 gene polymorphisms with risk of bone-joint and spinal tuberculosis: a meta-analysis

**DOI:** 10.1042/BSR20182217

**Published:** 2019-05-31

**Authors:** Wei Huang, Ruiyun Zhou, Jianfeng Li, Jianjun Wang, Hongwei Xiao

**Affiliations:** 1 Department of Spine and Osteology, Zhuhai People’s Hospital, Zhuhai Hospital of Jinan University, Zhuhai, Guangdong Province 519000, China; 2 Zhuhai Medical Emergency Center, Pre-Hospital Emergency Training Base Of Mid-southern China, Zhuhai, Guangdong Province 519000, China

**Keywords:** Bone tuberculosis, Meta-analysis, polymorphisms, TNF-α gene

## Abstract

The aim of the present study was to investigate the association of TNF-α-308 and TNF-α-238 gene polymorphisms with the risk of bone-joint and spinal tuberculosis (TB) by meta-analysis. By searching PubMed, Web of Science, Wanfang databases, CNKI, Medline, and Cochrane Library, the published articles about studies of the association of the TNF-α-308, TNF-α-238 gene polymorphisms with risk of bone-joint and spinal tuberculosis were collected by two reviewers. Begg’s and Egger’s tests were performed to assess publication bias. Stata 12.0 software was used for data analysis. The symmetry of the funnel plot indicated no significant publication bias in the Begg’s test (A: *P*=1.00, B: *P*=0.764), and the results of the Egger’s test showed no evidence of publication bias (A: *P*=0.954, B: *P*=0.626). Seven studies assessed the relationship between TNF-α-308 gene polymorphisms and risk of bone-joint and spinal tuberculosis risk. The heterogeneity (*I^2^*) of GG vs. AA or AG was 0% and there was no heterogeneity (χ^2^ = 0.06 and *P*=0.944) in a fixed-effects model. There was also a lack of association between TNF-α-308 polymorphism and bone-joint and spinal tuberculosis risk under the recessive model. The remaining models of the TNF-α-308 genotype and further studies of TNF-α-238 did not show a noteworthy association. Overall, there was no significant association between TNF-α-308, TNF-α-238 gene polymorphisms and bone-joint and spinal tuberculosis risk. Our study suggests that tumor necrosis factor α (TNF-α) gene polymorphisms may not contribute to bone-joint and spinal tuberculosis based on the current evidence.

## Introduction

Tuberculosis (TB) caused by *Mycobacterium tuberculosis* (Mtb) is an infectious disease that severely endangers human health in the world. Twenty-two countries in the world experience high morbidity due to tuberculosis, while China has the second largest incidence of tuberculosis [1]. Excluding pulmonary tuberculosis, osteoarticular tuberculosis is a common disease and the incidence of spinal tuberculosis is the highest [2]. Tumor necrosis factor α (TNF-α) plays an important role in the TNF-superfamily and it may be associated with a variety of organs and tissues’ physiological function or disease [3]. The physiological function of TNF-α depends on its concentration. Low concentrations cause infection, while high concentrations cause pathological damage. Besides, the sustained release of TNF-α will lead to cachexy but the appropriate dose could be immunological protection. There are four steps between TNF-α and anti-tuberculosis immunity: (i) Assist in the activation of macrophages; (ii) The formation and maintenance of tuberculous granuloma; (iii) The regulation of immune response; (iv) Regulation of osteoclast differentiation, activation and apoptosis [4].

TNF-α is a potent pleiotropic proinflammatory and immunoregulatory cytokine, which contributes to the initiation, up-regulation, and perpetuation of the inflammatory response. TNF-α is produced by many cell types including neutrophils, fibroblasts, NK cells, T and B cells, and macrophages infiltrating tissue as the part of host defense against brucellosis infection [5,6]. Increased levels of serum TNF-α has been observed in several infectious diseases including brucellosis [7,8], advanced tuberculosis [5], acute-phase Mediterranean spotted fever [6] and malaria. In conclusion, TNF-α may be an important factor in the early anti-inflammatory effects of infection, and it may have the necessary protective effect in the chronic phase of infection.

Studies have shown that TNF-α can induce osteoclast differentiation, activation and apoptosis directly or indirectly by binding to the corresponding receptor of osteoclast precursor cells [9]. Its production is dependent on TNF-α gene expression. The TNF-α gene polymorphism affects its expression of different genotypes which can produce different proteins affecting the susceptibility and severity of diseases. Evidence suggests TNF-a gene is associated with the development of tuberculosis [10]. There are some reports on the correlation between the TNF-α-308, TNF-α-238 gene polymorphism and tuberculosis infection in different populations, but the results are contrary [11,12]. There is no review that has attempted to synthesize the total available evidence of the role of TNF-α gene polymorphism in the pathogenesis of bone-joint and spinal tuberculosis. The current research on association of the TNF-α-308, TNF-α-238 gene polymorphisms with bone-joint and spinal tuberculosis risk was mainly single-center randomized controlled trial (RCT) or retrospective study with small sample size, and there is still lack of strict evidence-based medicine test. The purpose of the present paper is to investigate the association of the TNF-α-308 and TNF-α-238 gene polymorphisms with the risk of bone-joint and spinal tuberculosis by meta-analysis.

## Materials and methods

### Study collection

By searching PubMed, Web of Science, Wanfang databases, CNKI, Medline and Cochrane Library databases for articles published prior to January 2018, there were seven studies identified by two reviewers finally. The search strategy included using the keywords, such as ‘bone-joint & spinal tuberculosis’, ‘TNF-α’, ‘gene’, ‘polymorphisms’. To identify titles and abstracts of relevant literature, the reference that may have been missed during search or indexing were checked manually. (Supplementary material)

### Inclusion and exclusion criteria

All papers included in the meta-analysis met the following criteria: (i) investigating the association between TNF-α gene polymorphism and bone and joint tuberculosis, (ii) case-controlled studies and the controls had no malignant disease, (iii) the number of studies of genotypes must be completely provided in both experimental and control groups directly or indirectly. Exclusion criteria: (i) repeated studies; (ii) the control group did not meet the Hardy–Weinberg equilibrium (HWE) [13]; (iii) incomplete description of data or unclear sample data; (iv) animals experimental research; (v) summary, review etc.

### Data extraction

Data were extracted by two independent reviewers. Extraction of literature, including: first author, year of publication, country of study, method of gene detection, number of various genotypes. In the process of extraction, if there is a dispute, it is resolved through negotiation. Any disagreements on data extraction were resolved through discussion with other researchers.

### Quality evaluation

The quality of all research was assessed by the Newcastle–Ottawa Scale (NOS). The quality assessment for each research included the following aspects: (i) Queue selection; (ii) Comparability; (iii) Result measurement. According to the three aspects, the number of stars was divided. The lowest was 0 stars, the highest 10 stars, 0–3 stars were level C, the 4–6 stars were level B, and the 7–10 stars were level A [14]. If there was a disagreement in this process, it was decided by negotiation.

### Statistical analysis

Stata 12.0 software was used for meta-analysis as following: (1) Test of HWE. (2) Funnel plot analysis and Begg’s test, Egger’s test for publication bias. (3) Five different odds ratio (ORs) were computed: (i) G vs. A (allele model); (ii) GG vs. AA (homozygous model); (iii) GG vs. AA or AG (recessive model); (iv) GG or GA vs. AA (dominant model); and (v) GA vs. GG or AA (overdominance model). (4) Heterogeneity between studies was evaluated by chi-square-based Q test and *I*^2^ test. (*I*^2^ = 75–100%: extreme heterogeneity; *I*^2^ = 50–75%: large heterogeneity; *I*^2^ = 25–50%: moderate heterogeneity; *I*^2^ < 25%: no heterogeneity). If the *I*^2^ was lower than 50%, the pooled OR estimate of the study was calculated by a fixed-effects model. Otherwise, the random-effects model was used. (5) *P*<0.05 was considered statistically significant.

## Results

### Characteristics of studies

**Figure 1:**
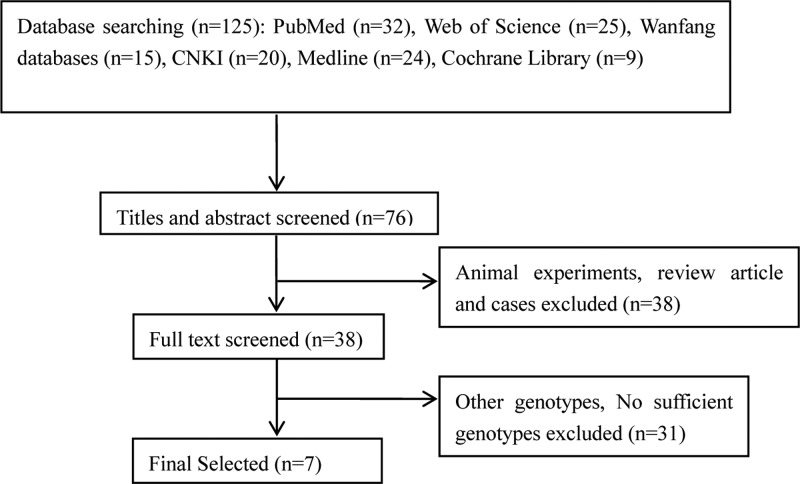
Literature search flow diagram

According to the above retrieval methods, a total of 125 relevant studies were selected. After skimming the titles, abstracts and reviewing the full text, 118 studies were excluded due to lack of detailed data or lack of data on target polymorphisms. Finally, seven case–control studies involving 1600 subjects were selected for the meta-analysis (Figure 1). The main characteristics of the eligible studies are presented in Table 1. The genotype and allele frequencies for each study as well as HWE of controls are summarized in Table 2. The quality of all research was assessed by the NOS in Table 3.

**Table 1:** Characteristics of the studies included in the meta-analysis

First author	Year	Country	Number	Age (years)	Gender (male %)	Genotyping method	Evaluated
			Cases/Controls	Cases/Controls	Cases/Controls		
Chunyan Lin	2011	China	46/40	39.2 ± 13.6/39.9 ± 7.4	63.0/42.5%	Bio-Rad Gel DOC 2000	TNF-α-308, 238
Dechun L.I. (1)	2015	China	81/55	42.6 ± 7.8/38.3 ± 10.2	54.3/65.5%	Bio-Rad Gel DOC 2000	TNF-α-308, 238
Dechun L.I. (2)	2015	China	65/50	39.2 ± 13.6/39.9 ± 7.4	50.1/54.0%	Bio-Rad Gel DOC 2000	TNF-α-308, 238
Y.J. Lv	2016	China	120/100	40.1 ± 8.5/NA	60.0/70.0%	Gel imaging system	TNF-α-308, 238
Yukun Zhang	2017	China	58/50	37.2 ± 13.2/39.3 ± 8.2	69.0/66.0%	NA	TNF-α-308, 238
Ying Zhou	2017	China	183/362	41.7 ± 18.0/45.1 ± 14.5	51.9/46.7%	Mass spectrometry platform	TNF-α-308, 238
Mingfeng Zheng	2018	China	240/150	NA	NA	Sanger sequencing method	TNF-α-308, 238

**Table 2:** TNF-α polymorphisms genotype distribution and allele frequency in cases and controls

TNF-α	Cases						Controls						HWE
	Total	GG (%)	GA (%)	AA (%)	G allele (%)	A allele (%)	Total	GG (%)	GA (%)	AA (%)	G allele (%)	A allele (%)	YES
**308**													
Chunyan Lin	46	43 (93.5)	3 (6.5)	0 (0)	89 (96.7)	3 (3.3)	40	40 (100)	0(0)	0 (0)	80 (100.0)	0 (0)	YES
Dechun LI(1)	81	70 (86.4)	10 (12.3)	1 (1.3)	146 (90.1)	16 (9.9)	55	38 (69.1)	14 (25.5)	3 (5.4)	107 (97.3)	3 (2.7)	YES
Dechun LI(2)	65	55 (84.6)	8 (12.3)	2 (3.1)	118 (90.8)	12 (9.2)	50	49 (98.0)	1 (2.0)	0 (0)	99 (99.0)	1 (1.0)	YES
Y.J. Lv	120	95 (79.2)	25 (20.8)	0 (0)	215 (89.6)	25 (10.4)	100	91 (91.0)	8 (8.0)	1 (1.0)	190 (95.0)	10 (5.0)	YES
Yukun Zhang	58	54 (93.1)	4 (6.9)	0 (0)	108 (93.1)	8 (6.9)	50	50 (100.0)	0 (0)	0 (0)	100 (100.0)	0 (0)	YES
Ying Zhou	183	170 (92.9)	13 (7.1)	0 (0)	353 (96.4)	13 (3.6)	362	333 (92.0)	27 (7.5)	2 (0.5)	693 (95.7)	31 (4.3)	YES
Mingfeng Zheng	240	217 (90.4)	23 (9.6)	0 (0)	457 (95.2)	23 (4.8)	150	127 (84.7)	23 (15.3)	0 (0)	277 (92.3)	23 (7.7)	YES
**238**													
Chunyan Lin	46	43 (93.5)	3 (6.5)	0 (0)	89 (20.7)	340 (79.3)	40	37 (92.5)	3 (7.5)	0(0)	77 (96.3)	3 (3.7)	YES
Dechun L.I. (1)	81	69 (85.2)	10 (12.3)	2 (2.5)	141 (87.0)	21 (13.0)	55	40 (72.7)	12 (21.8)	3 (5.5)	102 (92.7)	8 (7.3)	YES
Dechun L.I. (2)	65	55 (84.6)	10 (15.4)	0 (0)	120 (92.3)	10 (7.7)	50	48 (96.0)	1 (2.0)	1 (2.0)	97 (97.0)	3 (3.0)	YES
Y.J. Lv	120	87 (72.5)	26 (21.7)	7 (5.8)	200 (83.3)	40 (16.7)	100	82 (82.0)	13 (13.0)	5 (5.0)	177 (88.5)	23 (11.5)	YES
Yukun Zhang	58	51 (87.9)	7 (12.1)	0 (0)	112 (96.6)	4 (3.4)	50	46 (92.0)	4 (8.0)	0 (0)	96 (96.0)	4 (4.0)	YES
Ying Zhou	183	179 (97.8)	4 (2.2)	0 (0)	362 (98.9)	4 (1.1)	362	341 (94.2)	20 (5.5)	1 (0.3)	702 (97.0)	22 (3.0)	YES
Mingfeng Zheng	240	217 (90.4)	23 (9.6)	0 (0)	457 (95.2)	23 (4.8)	150	143 (95.3)	7 (4.7)	0 (0)	293 (97.7)	7 (2.3)	YES

**Table 3:** Quality evaluation of the included studies

Study	Queue selection	Comparability	Result measurement	Level of quality
Chunyan Lin	***	*	***	A
Dechun L.I. (1)	***	*	**	B
Dechun L.I. (2)	***	*	***	A
Y.J. Lv	****	*	***	A
Yukun Zhang	***	*	**	B
Ying Zhou	***	*	***	A
Mingfeng Zheng	***	*	***	A

### Meta-analysis results

**Figure 2:**
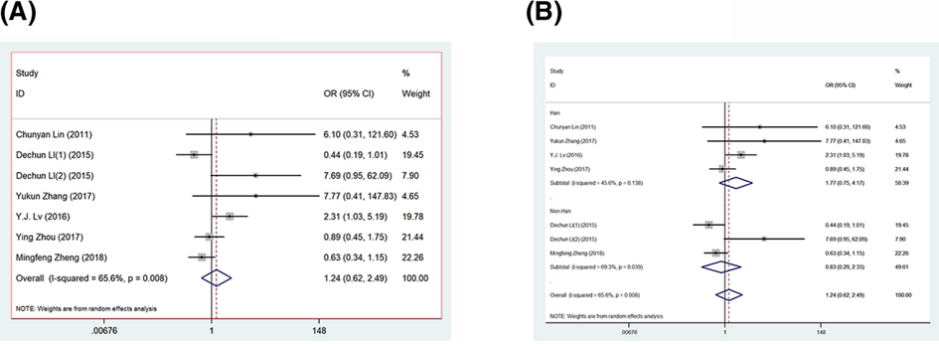
Forest plots (**A**) Forest plots of TNF-α-308 polymorphism and bone-joint and spinal tuberculosis risk based on recessive model (GG vs. AA or AG). (**B**) Forest plots of subgroups.

Seven studies assessed the relationship between TNF-α-308 gene polymorphisms and bone-joint and spinal tuberculosis (Figure 2) [15–21]. The heterogeneity of GG vs. AA + AG was assessed for all case–control studies. The *I*^2^ value was 0%, indicating no heterogeneity. Meta-analysis results were as follows: *χ*^2^ = 0.06 and *P*=0.944 in a fixed-effects model, indicating there was no association between TNF-α-308 polymorphism and bone-joint and spinal tuberculosis under the recessive model. Neither the remaining models of the TNF-α-308 genotype nor further studies in TNF-α-238 revealed a noteworthy association (Table 4).

**Table 4:** Meta-analysis of the association TNF-a polymorphisms and bone-joint and spinal tuberculosis risk

Polymorphisms	Comparison	Association				Heterogeneity	
		OR	95% CI	*P*-value	*I*^2^(%)	*P*-value	Model
TNF-a-308	G vs. A	0.982	0.886–1.087	0.721	0	0.995	F
	GG vs. AA	1.005	0.865–1.169	0.944	0	1.000	F
	GG vs. AA + AG	1.239	0.616–2.491	0.548	65.6	0.008	R
	GG + AG vs. AA	1.005	0.871–1.159	0.949	0	1.000	F
	GG + AA vs. GA	0.991	0.856–1.148	0.907	0	0.966	F
TNF-a-238	G vs. A	0.984	0.888–1.090	0.753	0	0.999	F
	GG vs. AA	1.004	0.864–1.166	0.963	0	1.000	F
	GG vs. AA + AG	1.215	1.054–1.401	0.007	12.2	0.337	F
	GG + AG vs. AA	0.769	0.319–1.855	0.559	0	0.739	F
	GG + AA vs. GA	0.980	0.847–1.134	0.786	0	0.989	F

Abbreviations: F, fixed-effects model; R, random-effects model; 95% CI, 95% confidence interval.

### Heterogeneity assessment and sensitivity analysis

Based on the results of heterogeneity test, the recessive model (GG vs. AA or AG) of TNF-α-308 (*I*^2^ = 65.6%, *P*=0.008) indicated obvious heterogeneity in *I*^2^ test and its pooled OR estimate of the study was calculated by the random-effects model. The same was true for the recessive model (GG vs. AA + AG) of TNF-α-238 in the *I*^2^ test (*I*^2^ = 83%, *P*=0.000). The other *I*^2^ test (*I*^2^ = 0, *P*>0.05) of genotype indicated no obvious heterogeneity, and the fixed-effects model was used. One study with small number of cases was eliminated for sensitivity analysis, and the other six studies were analyzed again. The results showed that lack of cases still had no effect on the results of meta-analysis in the six studies.

### Publication bias

**Figure 3:**
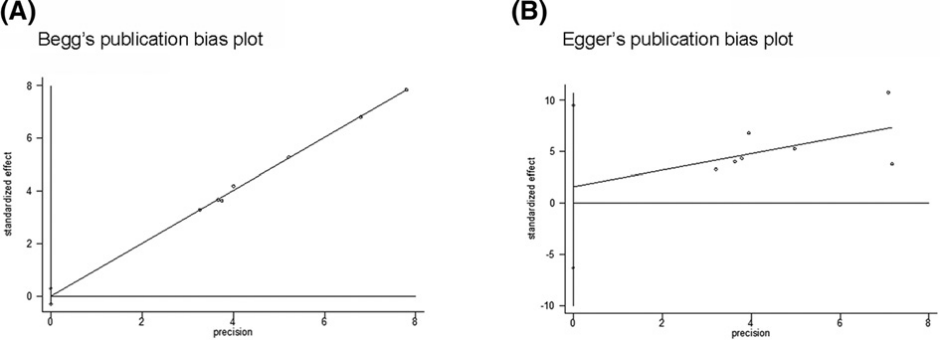
Begg’s test and Egger’s test were performed to evaluate the publication bias

Begg’s test and Egger’s test were performed to evaluate the publication bias (Figure 3A,B). Symmetry of the funnel plots suggested no obvious publication bias in Begg’s test (A for TNF-α-308: *P*=1.00, B for TNF-α-238: *P*=0.764), and the results of Egger’s test suggest no evidence of publication bias (A: *P*=0.954, B: *P*=0.626).

## Discussion

Our study showed that there was a lack of association between TNF-α-308 polymorphism and bone-joint and spinal tuberculosis risk under the recessive model. The remaining models of the TNF-α-308 genotype and further studies of TNF-α-238 did not show a noteworthy association. There was no significant association between TNF-α-308, TNF-α-238 gene polymorphisms and bone-joint and spinal tuberculosis risk.

Bone-joint tuberculosis is a common infectious disease in the world, which is common in children and adolescents. However, with the increase in life expectancy, the probability of bone-joint tuberculosis in the elderly has also been greatly increased [22]. Bone-joint tuberculosis is a secondary tuberculosis whose primary lesion is pulmonary tuberculosis or digestive tract tuberculosis. In the active stage of primary lesion, Mtb reaches the bone or joint through blood circulation while it does not necessarily happen immediately. It can be latent for many years in the bone or joint, until in the situation of low immunity, such as injury, malnutrition, excessive fatigue, diabetes, major operation and other inducing factors, which can lead the latent Mtb to be active and appearance of clinical symptoms. The most common site of bone-joint tuberculosis is the spine, accounting for 50%, followed by knee joint, hip joint and elbow joint [23]. Therefore, it can be seen that the location where the bone and joint tuberculosis is located has a great negative impact on the activity and is prone to trauma. The first pathological change that occurs is exudative inflammation of the bone and joint tuberculosis, followed by hyperplastic or necrotizing lesions. Bone and joint tuberculosis can be divided into three types: simple-synovial tuberculosis, simple-bone tuberculosis and total-joint tuberculosis. Simple bone tuberculosis is common. If the early stage of tuberculosis is well controlled, the joint function will not be affected remarkably. But when the lesion develops further, the tuberculosis will perforate the articular surface, enter the joint cavity, and lead the articular cartilage surface to be damaged to varying degrees finally. This is called total joint tuberculosis which will lead to varying degrees of joint dysfunction.

The susceptibility to TB depends upon different factors and the risk of developing diseases after infection with Mtb ranges from 5 to 10%. This suggests that besides the mycobacterial itself, the host genetic factors may determine the differences in host susceptibility to TB [24–26]. Among the important risk factor, cytokines and specially TNF-α genes, are thought to be responsible in regulating the protective immune responses [27–31]. TNF-α, gene that encodes the cytokines TNF-α is located within the class III region of the MHC [32]. TNF-α is expressed as a transmembrane protein that can be processed into a soluble form (sTNF) [33] that exerts its functions via two receptors TNF receptor 1 (TNFR1) and TNFR2. The former exists in almost all cells aims to trigger apoptosis signaling pathways while the latter restricts expression and activation of cell survival.

There are four steps between TNF-α and anti-tuberculosis immunity [34]: (i) TNF-α can activate macrophage which promotes the formation and maturation of phagolysosome, and stimulates the production of reactive nitrogen intermediates by cooperating with IFN, controlling or killing Mtb, enhance macrophage and DC antigen presentation function in order to activate T cells, and promote the production of IFN cytokines. (ii) Promote the formation of tuberculous granuloma which can seal the infection off, limit the spread of bacteria, protect the surrounding tissues, and provide a range of interactions for immune cells and cytokines, so as to enhance the bactericidal effect. (iii) Antagonizes inflammation, limits lesions and tissue damage. (iv) Contribute to combination with the corresponding receptors on the osteoclast precursor cells, directly or indirectly regulating the differentiation, activation and apoptosis of the osteoclast cells in the whole process.

TNF-α is encoded by the TNF-α gene, and its expression is mainly focused on the promoter region of the TNF-α gene [35]. The single nucleotide polymorphisms (SNPs) in this region affect TNF-α expression levels and regulate body’s resistance and susceptibility to tuberculosis infection. The gene polymorphisms related to bone-joint tuberculosis include vitamin D receptor gene (VDR), interferon and its receptor gene (IFN-γ), monocyte chemoattractant protein 1 (MCP-1) gene, human leukocyte antigen (HLA) gene and TNF-α gene. Currently, there were some researches on the relationship between TNF-α gene polymorphism and tuberculosis, but the results were contrary. Studies were focused on the relationship between TNF-α gene polymorphisms and bone and joint tuberculosis in different regions, races and ethnicities. Most of the results indicated that TNF-α-308 and TNF-α-238 genes had a certain correlation. Olsen, found that genetic polymorphism was associated with bone-joint tuberculosis [36]. He showed that TNF-α gene 308G/A could increase the risk of pulmonary tuberculosis which was first discovered by Pantelidis et al. [37], but Selvaraj et al. [38] and Vejbaesya et al. [39] had not found that there was an association between TNF-α gene polymorphism and bone-joint tuberculosis in Indian and Thai populations.

In the present study, four randomized controlled trial (RCT) showed that bone-joint tuberculosis was associated with TNF-α-308 and TNF-α-238 genetic polymorphisms, and allele ‘A’ may play a protective role. One study showed that bone-joint tuberculosis was associated with TNF-α-308 genetic polymorphisms, and allele ‘A’ played a protective role but no obvious genetic polymorphisms with TNF-α-238. Only two studies showed that bone-joint tuberculosis was not associated with genetic polymorphisms of TNF-α -308 or 238. The main contribution of this meta-analysis is to systematically evaluate the TNF-α gene polymorphism in patients with bone-joint tuberculosis, and to draw a comprehensive conclusion. Our analysis suggested that there was no correlation between TNF-α-308 or TNF-α-238 gene polymorphisms and bone-joint tuberculosis. This conclusion is similar to the research pulmonary tuberculosis by Sharma et al. [40] and Vejbaesya et al. [39].

However, the results of this analysis are subject to large samples, multicenter collaboration and further randomized controlled trials to validate. Our study does not imply that all polymorphisms in the TNF-α locus are completely unrelated to the susceptibility of bone and joint tuberculosis. Gene–environment interactions might also affect the results.

There were also some limitations in our study. Our research focuses on the Chinese population. Non-Chinese studies were not excluded from this review, and according to our inclusion/exclusion criteria, only Chinese populations were finally included. In the future, we should analyze the correlation between bone and joint tuberculosis and TNF-α gene polymorphism in various groups. Therefore, more scholars are expected to explore more genes related to tuberculosis susceptibility, promote the development of tuberculosis gene therapy, and benefit more patients with tuberculosis. Further study on subgroup analysis should be performed based on ethnicity, countries, regions, type of control source and so on. Moreover, the study may be influenced by false-positives, gene–environment interactions and longitudinal studies were needed to infer the causation.

## Supporting information

**Supplementary Material 1:** Search Strategy Used in All Databases

## Abbreviations

HWE: Hardy–Weinberg equilibrium
Mtb: *Mycobacterium tuberculosis*
NOS: Newcastle–Ottawa Scale
OR: odds ratio
RCT: randomized controlled trial
TB: tuberculosis
TNF-α: tumor necrosis factor α
