# Supplementary material for: Association of the TNF-α-308, TNF-α-238 gene polymorphisms with risk of bone-joint and spinal tuberculosis: a meta-analysis
Source: Biosci Rep. 2019 May 31;39(5):BSR20182217. doi: 10.1042/BSR20182217 (PMC6542758; doi:10.1042/BSR20182217)
Supplement: Supplementary file 1 [file bsr20182217_Supp1.pdf]

Supplementary Material 1. Search Strategy Used in All Databases

Medline via PubMed

	Searches	Results
#1	bone tuberculosis OR spinal tuberculosis OR tuberculosis OR TNF-a gene OR gene OR polymorphisms	2763
#2	“risk” OR “influence” OR “relationship”OR “effect”OR “relevance”	2468
#3	#1 AND #2	**56**

EMBASE via Elsvier

	Searches	Results
#1	bone tuberculosis OR spinal tuberculosis OR tuberculosis OR TNF-a gene OR gene OR polymorphisms	186

#2	“risk” OR “influence” OR “relationship”OR “effect”OR “relevance”	3949
#3	#1 AND #2	**0**

CENTRAL

	Searches	Results
#1	(bone tuberculosis OR spinal tuberculosis OR tuberculosis OR TNF-a gene OR gene OR polymorphism s):ti,ab,kw	55
#2	MeSH descriptor: [relationship] explode all trees	112
#3	(“risk” OR “influence” OR “relationship”OR “effect”OR “relevance”):ti,ab,kw	233
#4	#1 AND (#2 OR #3)	**0**

CINAHL via EBSCO

	Searches	Results
#1	bone tuberculosis OR spinal tuberculosis OR tuberculosis OR TNF-a gene OR gene OR polymorphisms	27
#2	“risk” OR “influence” OR “relationship”OR “effect”OR “relevance”	207
#3	#1 AND #2	**0**

AMED via EBSCO

	Searches	Results
#1	bone tuberculosis OR spinal tuberculosis OR tuberculosis OR TNF-a gene OR gene OR polymorphisms	120
#2	“risk” OR “influence” OR “relationship”OR “effect”OR “relevance”	310
#3	#1 AND #2	**0**

Cochrane Library

	Searches	Results
#1	bone tuberculosis OR spinal tuberculosis OR tuberculosis OR TNF-a gene OR gene OR polymorphis ms	566
#2	“risk” OR “influence” OR “relationship”OR “effect”OR “relevance”	758
#3	#1 AND #2	**9**

Web of Science

	Searches	Results

#1	bone tuberculosis OR spinal tuberculosis OR tuberculosis OR TNF-a gene OR gene OR polymorphisms	899
#2	“risk” OR “influence” OR “relationship”OR “effect”OR “relevance”	1113
#3	#1 AND #2	**25**

CNKI

	Searches	Results
#1	(SU='骨结核'+'脊柱结核'+'结核'+'肿瘤坏死因子a基因'+'肿瘤坏死因子a基因'+'多态性'因'+bone tuberculosis'+'spinal tuberculosis'+'tuberculosis'+'TNF-a gene'+'gene'+'polymorphisms') AND (SU='骨结核'+'基因')	**20**

Wanfang data

	Searches	Results
#1	('骨结核'+'脊柱结核'+'结核'+'肿瘤坏死因子a基因'+'肿瘤坏死因子a基因'+'多态性'因'+bone tuberculosis'+'spinal tuberculosis'+'tuberculosis'+'TNF-a gene'+'gene'+'polymorphisms') * ('骨结核'+'基因')	**15**

VIP

	Searches	Results

#1	题名或关键词=骨结核 或者 题名或关键词=脊柱结核 或者 题名或关键词结核 或者 题名或关键词=肿瘤坏死因子a基因 或者 题名或关键词=多态性 或者 题名或关键词=bone tuberculosis 或者 题名或关键词=针刀 或者 题名或关键词=spinal tuberculosis题名或关键词=tuberculosis 或者 题名或关键词=TNF-a gene 或者 题名或关键词=gene题名或关键词=polymorphisms	**0**

OASIS

	Searches	Results
#1	뼈결핵 AND 유전자 다형성	**0**

KCI

	Searches	Results
#1	뼈결핵 AND 유전자 다형성	**0**

RISS

	Searches	Results
#1	뼈결핵 AND 유전자 다형성	**0**
